# High-expression of Galactosidase alpha is correlated with poor prognosis and immune infiltration in low-grade glioma

**DOI:** 10.7150/jca.81975

**Published:** 2023-03-05

**Authors:** Yi Zhang, Jinchuan Li, Xiaofeng Yin

**Affiliations:** Department of Neurosurgery, Second hospital of Shanxi Medical University, China

**Keywords:** Low-grade glioma, Galactosidase alpha, Prognosis, Immune infiltration

## Abstract

**Background:** Galactosidase alpha (GLA), a member of galactosidase (GAL) family, contributes to cancer diagnosis and targeted therapy. Up to now, neither prognosis nor immune infiltration has been demonstrated in cases with low-grade glioma (LGG). In LGG, we investigated the association between GLA expression and immune infiltration levels.

**Methods:** GLA expression levels in pan-cancer were evaluated utilizing the Oncomine database. In addition, GLA level was screened via analyzing the gene expression omnibus (GEO) data and the Cancer Genome Atlas (TCGA) data, and evaluated in LGG tissues and adjacent tissues by using qPCR. TIMER database was utilized for evaluating the correlation between GLA level and LGG immune infiltrates. A correlation was found between GLA levels and LGG immune infiltrates utilizing the TIMER database. Moreover, we then assessed the TIMER data to explore clinical outcome in multiple immune cells and the correction between GLA expression and immune markers.

**Results:** The mRNA levels of GLA were upregulated in LGG tissues. GLA expression was associated with a poor outcome of patients with LGG. Additionally, the infiltration levels of several immune cells were obviously enriched in LGG with a higher GLA level. Moreover, LGG prognosis was worsened with high GLA levels in immune cells.

**Conclusions:** These results suggested that GLA levels in LGG might be more predictive of immune infiltration, with potential value for assessment of tumor development.

## Introduction

The most common intracranial tumor is glioma, a grave health issue in the central nervous system (CNS) 1-3 and the most considerable matter of tumor incidence or tumor-related deaths 4-6. In spite of development in tumor diagnosis and therapies, about 50% of cases with low-grade glioma (LGG), which consist of grade II-III tumors, suffer from metastatic disease 7. Approximately 40% of patients survive within five years of progression-free survival (PFS) 8. For non-metastasized LGG, total resection is an occasionally curative therapy 9, and antiepileptic drugs are also administered for control of tumor process 10. However, the malignant transformation based on a higher histological grade to patients initially diagnosed with LGG is a well-recognized phenomenon 11. There is an urgent requirement of recognized novel biomarkers that allow impeding the progression of LGG to high-grade. It will be a benefit for resection surgery and neo-adjuvant treatments for LGG management.

Tumor microenvironment that consists of inflammatory cytokines, immune cells and stromal cells has been reported to be an important regulator of tumor progression and recurrence 12. In this regard, infiltrating by immune cells, including T cells, natural killer (NK) cells, macrophages, dendritic cells (DC), B cells and macrophages, is significantly associated with tumor malignancy and patient outcome 13. The role of immune cells has been well-established in tumor immune escape of several malignancies 14. Data suggested that an increasing of T CD4^+^ memory cells or NK cells seems to be a predisposing factor for worse outcome in patients with GBM 15. A significant infiltrating level of macrophages in glioma has been recently suggested as an essential mechanism for immune suppressive phenotype 13. Up to now, there has been no detailed investigation of biomarkers which are reliably used for detecting immune infiltration levels of LGG tumors to provide a novel immunotherapeutic target.

The ubiquitous and conserved galactosidase alpha (GALA or GLA), a member of galactosidase (GAL) family, encodes a glycoprotein hydrolyzing the terminal alpha-galactosyl moiety from glycoconjugates. A study has proved the importance of lysosomal exoglycosidases, including β-hexosaminidase, β-galactosidase and α-mannosidase, which are increased in glioma tissues and exert influences on promoting cancer cell infiltration from primary tumors and locally aggressive characteristic 16. In addition, GLA expression in thyroid carcinoma tissues is shown to contribute to galactose metabolism pathway and is potentially used as a cancer diagnostic marker or therapeutic target 17. Moreover, GLA deficiency can activate NK cells in wild-type mice 18. Till now, neither GLA expression nor immune infiltration has been demonstrated to correlate with LGG. An analysis of prospective data was performed in this study to explore whether GLA level in LGG patients could predict overall survival (OS) and immune cell infiltrating.

## Methods

### Data source

The clinical and pathological data of TCGA pan-cancer were acquired from the Cancer Genome Atlas (TCGA, www.cancer.gov/about-nci/organization/ccg/research/structural-genomics/tcga). We utilized Oncomine database to assess GLA expression levels in pan-cancer 19. Moreover, GSE4290 and GSE16011 datasets were obtained from the National Center of Biotechnology information (NCBI, www.ncbi.nlm.nih.gov/). GEO2R online tool was used for analyzing GLA expression in LGG group and normal group.

### Human Tissue Samples

We collected twenty-three samples of LGG in the Second hospital of Shanxi Medical University, China. The Ethics Committee for Clinical Trials of Second Hospital of Shanxi Medical University approved this research. The detailed including criteria for the enrolled patients in this study are: (1) all the patients were diagnosed LGG with WHO criteria 20; (2) patients did not have other tumors or diseases; (3) patients did not receive radiation treatment and chemotherapy drugs; (4) patients received MRI examination within three days before and after tumor resection. Meanwhile, the adjacent brain tissues were obtained from patients during surgery. The clinical parameters were shown in** Table 1**. All the samples were immediately frozen in liquid nitrogen and prepared for RNA extraction.

### qPCR

Total RNAs of LGG samples and adjacent tissues were extracted by utilizing TRIzol solutions (Invitrogen, Carlsbad, CA, USA). Reverse transcription was carried out utilizing SuperScript™ first-chain Synthesis reagent (Invitrogen). The obtained cDNAs were subjected to real-time quantitative PCR. A total of 20 μl reaction was prepared with 10 μL qPCR Master Mix (2X), 1 μg of cDNA templates, 1 μL of forward and reverse primer. Real-time PCR cycles were performed on a Bio-Rad iQ5 system. The quantification of GLA levels in LGG was calculated with ΔΔCt method. The statistic difference between LGG group and adjacent group was assessed utilizing Student's T test. It was considered as significance when P < 0.05 is present.

### Gene expression Profiling Interactive Analysis 2 (GEPIA2) analysis

In this research, we utilized GEPIA2 online tool (http://gepia.cancer-pku.cn/) to assess the correlation between GLA expression level and overall survival rate in pan-cancer. We selected GEPIA “Survival Plots” module and entered the gene name “GLA”. The cutoff between low and high GLA level group is 50%. The Cox PH Model was used to calculate the hazards ratio in GEPIA2.

### TIMER immune infiltration analysis

An immune infiltrate level measurement was conducted in several immune cells using the Tumor Immune Estimation Resource (TIMER) “Immune-Gene” module. We entered “GLA” as gene of interest. The TIMER tool visualized the correlation of GLA expression with immune infiltration level in diffuse tumor types. We selected the “Purity Adjustment” button to counteract the major confounding factor in association analysis. And then the scatter plots showed the relationship between infiltrates estimation value and GLA expression level with the purity-adjusted spearman's rho in LGG. In addition, TIMER “Immune-Outcome” module was utilized to assess the clinical relevance of tumor immune. The infiltration was divided into low level (50%) and high level (50%). In all cases, partial spearman's rho (purity-adjusted) coefficients and correlation (cor) were calculated through CIBERSORT algorithm. Kaplan-Meier curves displayed the hazard ratios for Cox model and the log-rank p values.

## Results

### GLA expression analysis across pan-cancer and LGG

**Figure 1:**
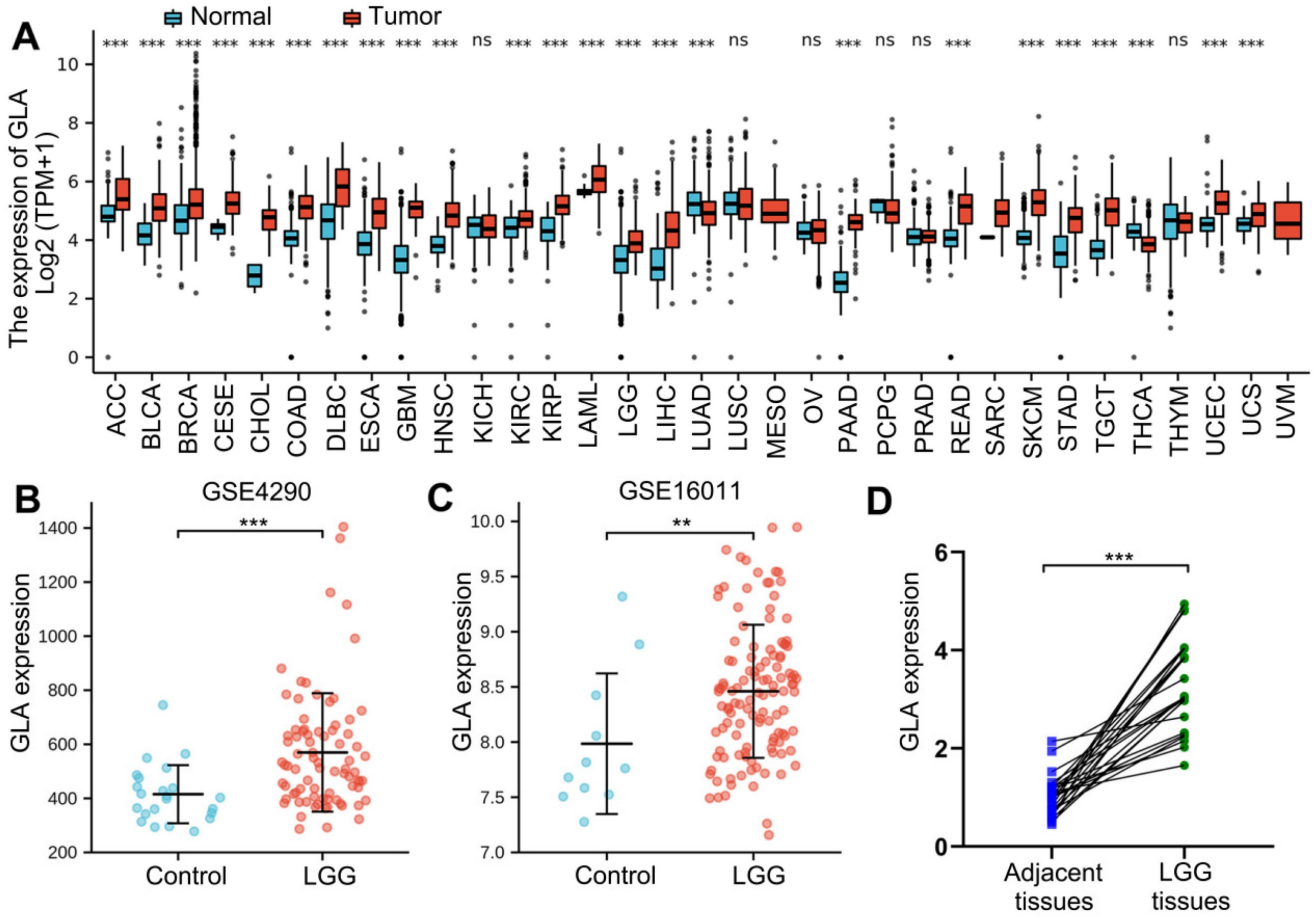
** GLA expression analysis across pan-cancer and LGG.** (A) Different expression levels of GLA between multiple tumor tissues and normal tissues by analyzing TCGA data. (B-C) Verification of GLA mRNA expression levels in LGG GSE4290 and GSE16011 dataset. (D) Scatter diagram of GLA mRNA levels in twenty-three pairs of LGG tissues and adjacent samples. **P<0.01, ***P<0.001.

Firstly, to assess GLA expression level in pan-cancers, TCGA data involved several tumors were analyzed with Oncomine online tool. We analyzed the molecular profiles of 33 tumor types. The normal specimens served as individual control. Among the 33 tumor types investigated, 22 tumor types expressed more GLA, compared with TCGA normal tissue samples (**Figure 1A**). Moreover, severely increased levels observed in both of LGG and GBM tissues (**Figure 1A**). While lung adenocarcinoma (LUAD) and thyroid carcinoma (THCA) showed significantly decreased mean GLA expression in comparison with normal tissues (**Figure 1A**). For further GLA expression analysis, we selected two GSEA datasets that included brain tissue subjects and patients with LGG. The data exhibited that the high expression levels of GLA in LGG groups of GSE4290 dataset (P<0.001, **Figure 1B**) and GSE16011 dataset (P<0.01, **Figure 1C**). We further examined GLA mRNA levels in human LGG tissues. qRT-PCR analysis displayed that 74% (17/23) of LGG tissue samples significantly showed increased GLA levels compared to adjacent specimens (**Figure 1D**). According to these data, LGG tissues expressed high levels of GLA, implying that GLA might be involved in the malignance of LGG.

### Prognostic value of GLA level across pan-cancer and LGG

**Figure 2:**
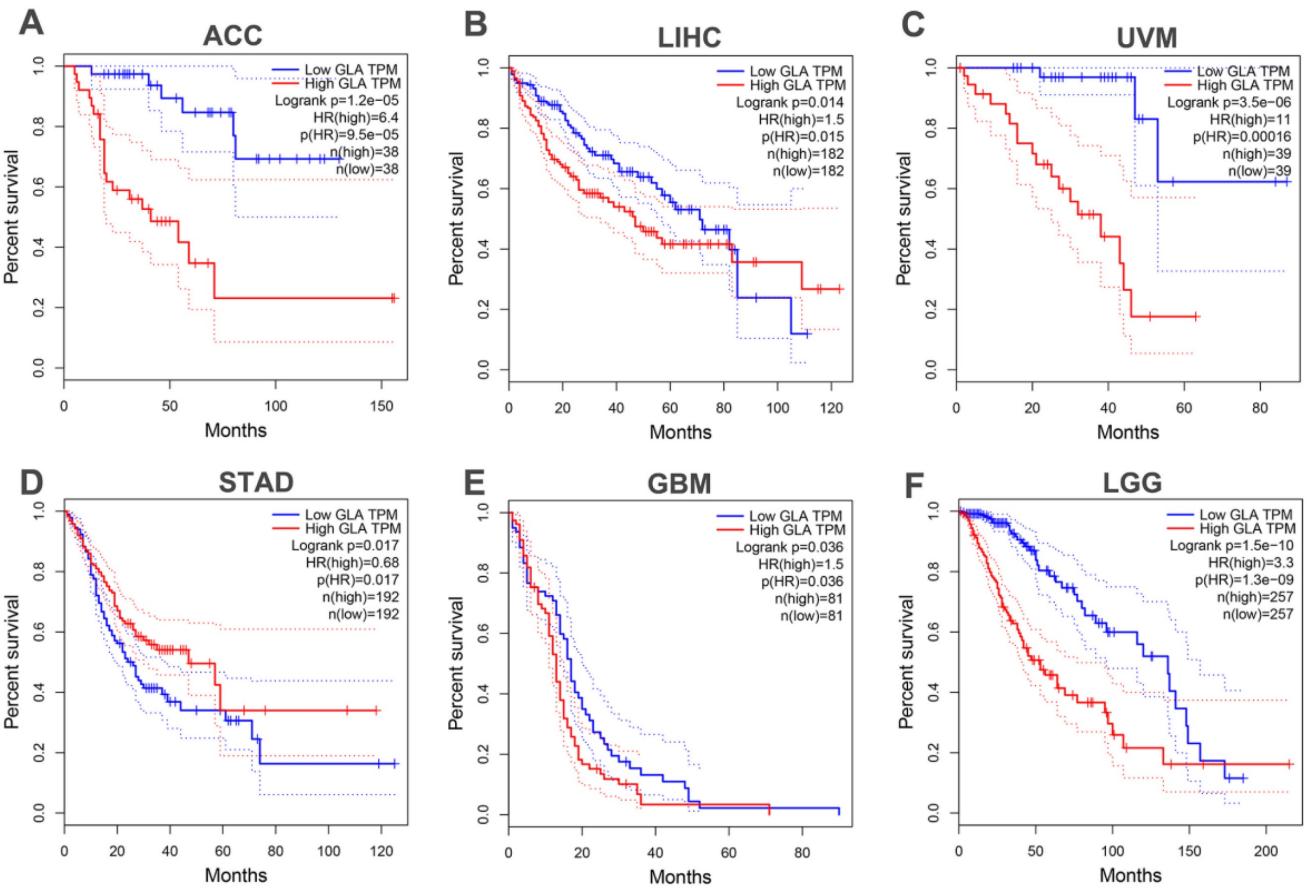
** Prognostic value of GLA level across pan-cancer and LGG.** High expression level of GLA is related to worse overall survival in patients with ACC, LIHC, UVM, STAD, GBM or LGG. Samples with high GLA expression levels was labeled as red curve. Samples with low GLA expression levels was labeled as blue curve. HR, hazard ratio; TPM, Transaction per million.

Next, in the TCGA database, we analyzed survival data for patients with pan-cancer. It was found that OS of patients with adrenocortical carcinoma (ACC) (P<0.001, **Figure 2A**), liver hepatocellular carcinoma (LIHC) (P=0.015, **Figure 2B**) and uveal melanoma (UVM) (P=0.001, **Figure 2C**) was obviously shorter in GLA overexpression group than in the GLA downexpression group. However, **Figure 2D** revealed that low GLA expression correlated significantly with worse OS in stomach adenocarcinoma (STAD) (P=0.017) cohort. Moreover, we validated the data in GBM and LGG cohorts, indicating a significant prolongation of the OS time in GBM (P=0.036, **Figure 2E**) or LGG (P<0.001, **Figure 2F**) patients with low expression level of GLA relative to patients with high GLA level. These data indicated that high GLA level was related to worse outcome in LGG.

### GLA is associated with immune cell infiltration

**Figure 3:**
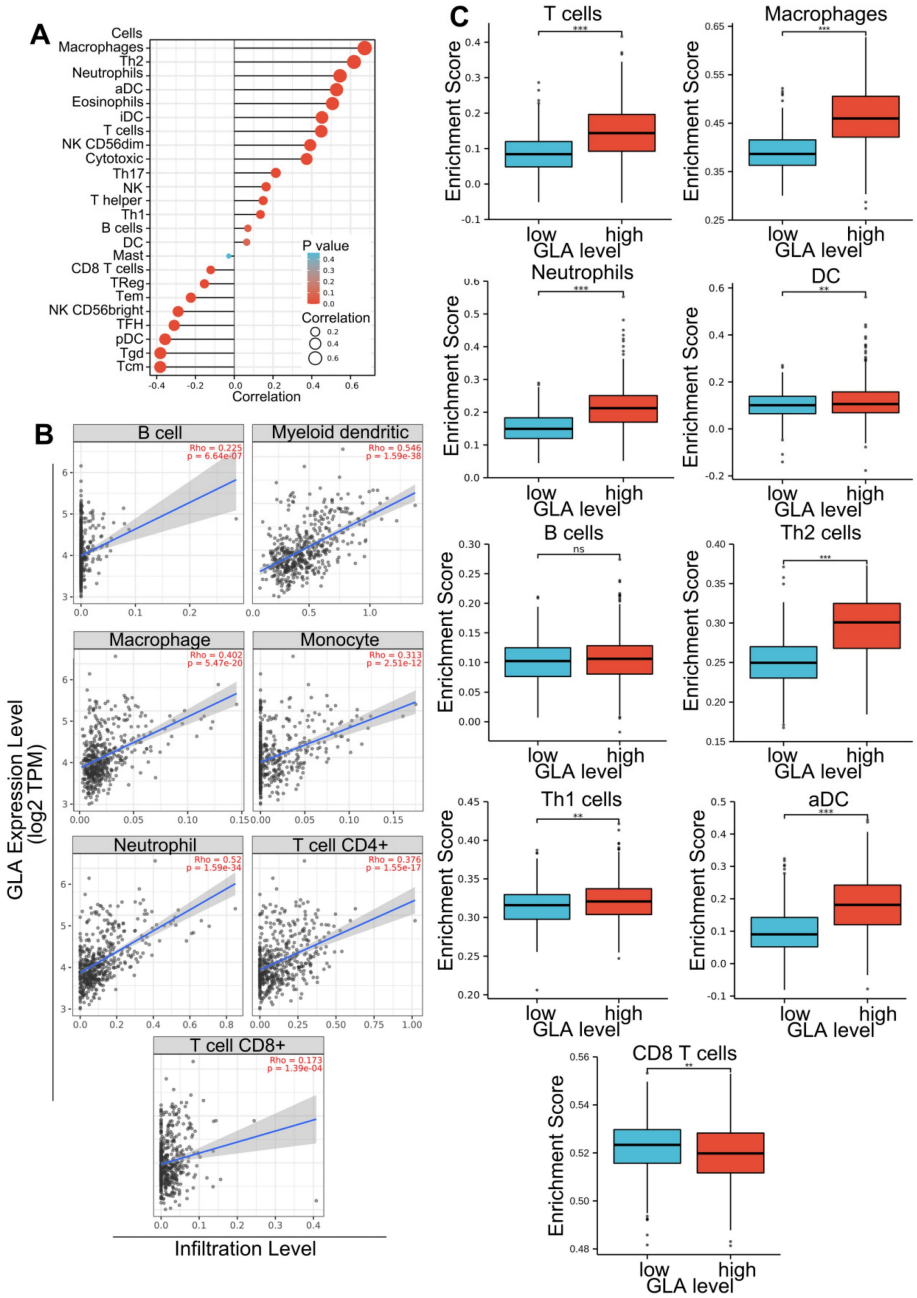
** GLA is associated with immune cell infiltration.** (A-B) Correlation of GLA expression level with multiple immune cell infiltration levels in LGG. The immune cells we analyzed were B cells, myeloid dendritic cells, macrophages, monocyte, neutrophils, CD4^+^ T cells and CD8^+^ T cells. (C) Enrichment score of multiple immune cells in low GLA expression group and high GLA expression group. P<0.01, P<0.001.

Gene expression of LGG patients in TCGA-LGG project was subjected to the assessment of immune cell infiltration levels. Firstly, integrate correlation analysis was conducted between principal variables and immune infiltration matrix data in the TCGA-LGG project. The analysis results were visualized with the lollipop chart using ggplot2 package. The correlations between immune infiltrates and GLA levels were evaluated using Spearman's test. As shown in **Figure 3A**, GLA expression was positively correlated with eight types of immune cells, including aDC, macrophages, Th2 cells, eosinophils, neutrophils, T cells, cytotoxic cells and T helper cells in LGG (Spearman's correlation > 0.3). In addition, GLA expression was negatively correlated with pDC and NK CD56bright cells. We found the positive association between GLA level and infiltration in B cell (Rho=0.225), myeloid dendritic cell (Rho=0.546), macrophage (Rho=0. 402), monocyte (Rho=0.313), neutrophil (Rho=0.52), T cell CD4^+^ (Rho=0.376) and T cell CD8^+^ (Rho=0.173) (P<0.001, **Figure 3B**). Moreover, we used ssGSEA immune cell algorithm to evaluate enrichment score of immune cells in GLA low and GLA high groups. A significant increase of infiltration level in T cells, macrophages, neutrophils, DC, Th2 cells, Th1 cells, and aDC was found in the high GLA group (P<0.01, P<0.001, **Figure 3C**). The results indicated that GLA might perform an increasingly important role in immune infiltrate of LGG, especially in the infiltration of neutrophils, macrophages and myeloid dendritic cells.

### Survival analysis of the association between clinical outcome and immune infiltrates

**Figure 4:**
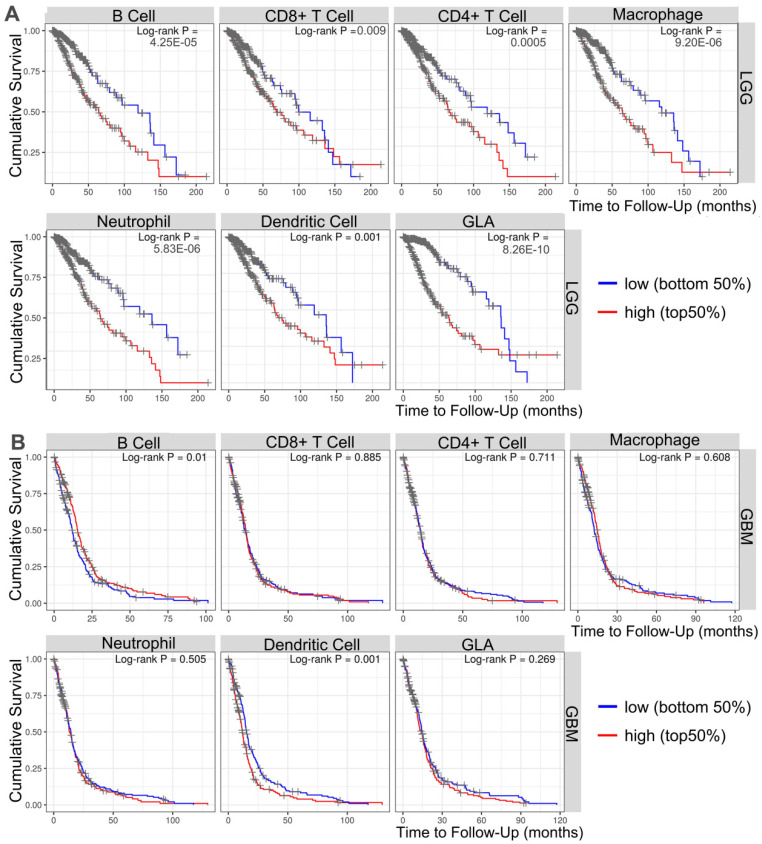
** Survival analysis of the association between clinical outcome and immune infiltrates.** The results from TIMER database showing the correlation between GLA expression levels and infiltrating levels of B cells, CD8^+^ T cells, CD4^+^ T cells, macrophages, neutrophils, and dendritic cells in (A) LGG and (B) GBM samples.

To investigate the important relevance between GLA expression and immune infiltration levels in LGG and GBM, the immune infiltrates analysis was performed by using TIMER. TIMER is an integrated database for comprehensive analysis of immune infiltrates across multiple tumor types. These data in **Figure 4A** showed that the high infiltrates levels of all six immune cells (B cell, CD4^+^ cells, CD8^+^ cells, macrophages, neutrophils and dendritic cells) were associated with poor prognosis of LGG cases (P<0.001). More precisely, the poor survival was obviously related with high infiltrates levels in B cells (log-rank P = 4.25e-05), CD8^+^ T cells (log-rank P = 0.009), CD4^+^ T cells (log-rank P = 0.0005), macrophages (log-rank P = 9.20e-06), neutrophils (log-rank P = 5.83e-06) and dendritic cells (log-rank P = 8.26e-10). Moreover, two types of tumor-infiltrating immune cells, including B cells (log-rank P = 0.01) and dendritic cells (log-rank P = 0.001) were obviously related to the prognosis in GBM patients (**Figure 4B**). The data indicated that it is possible that LGG level influences prognosis by modulating tumor-infiltrating immune cells.

### Correlation analysis of GLA expression and immune infiltration markers

**Figure 5:**
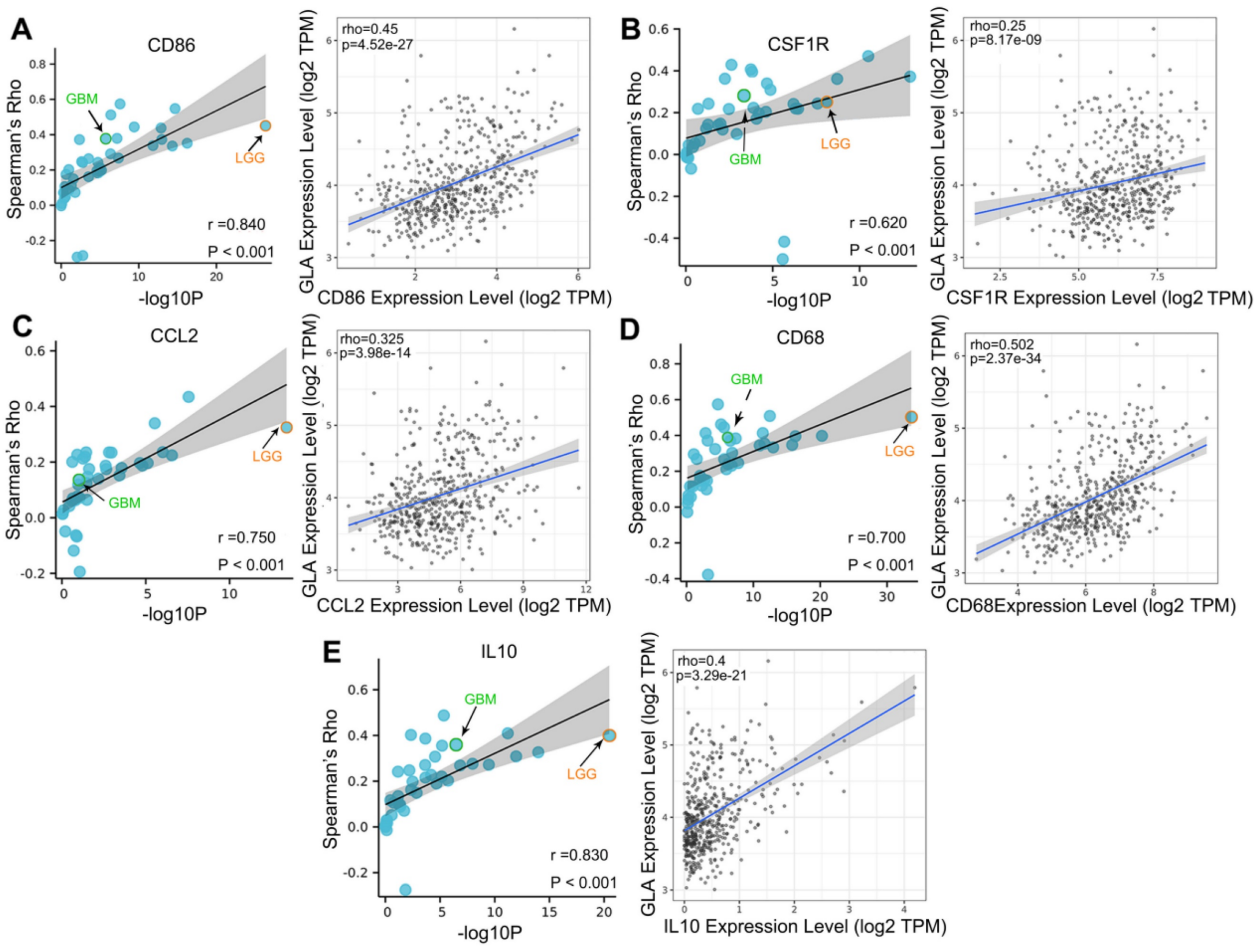
** Correlation analysis of GLA expression and immune infiltration markers in pan-cancer.** Correlation between GLA level and multiple immune-related markers, including (A) CD86, (B) CSF1R, (C) CCL2, (D) CD68 and (E) IL10 in pan-cancer. Each blue circle represents a type of tumor. GBM sample was marked as green circle. LGG sample was marked as orange circle.

**Figure 6:**
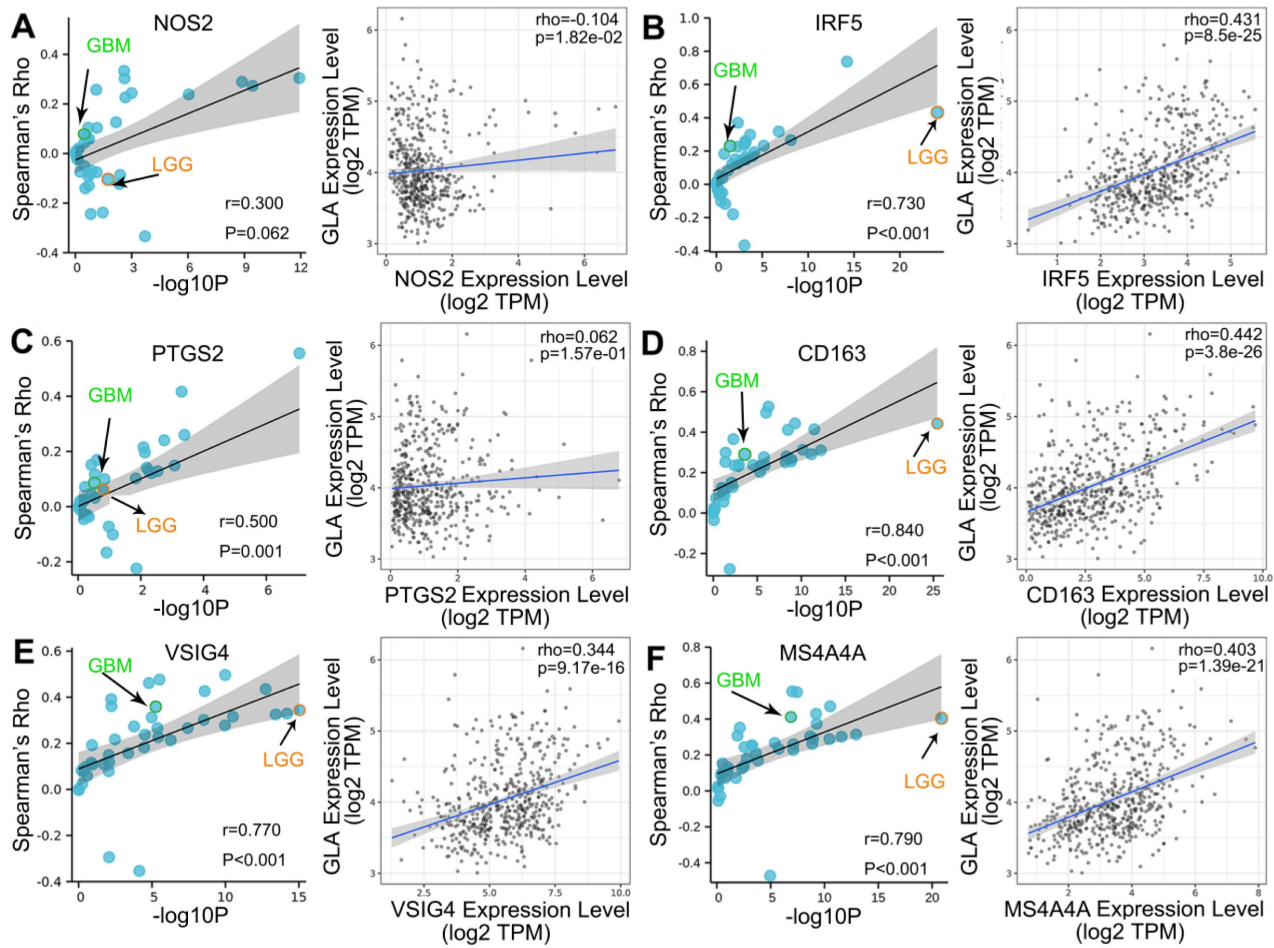
** Correlation analysis of GLA expression and immune infiltration markers.** Correlation between GLA level and multiple immune-related markers, including (A) NOS2, (B) IRF5, (C) PTGS2, (D) CD163, (E) VSIG4 and (F) MS4A4A in pan-cancer. Each blue circle represents a type of tumor. GBM sample was marked as green circle. LGG sample was marked as orange circle.

We explored the co-expression of GLA with key immune markers to assess the correlation between GLA and immune infiltration. **Table 2** displayed the correlations between GLA expression and 56 kinds of gene markers involved T cell, B cell, monocyte, macorphages, neutrophils, natural killer cell, dendritic cell and so on. In LGG, the correlation with GLA expression was stronger for T cell markers (CD2, Rho=0.518, P<0.001; CD3D, Rho=0.479, P<0.001), TAM markers (CD68 Rho=0.502, P<0.001), M2 macrophage markers (CD163, Rho=0.442, P<0.001) and dendritic cell markers (HLA-DPB1, Rho=0.572, P<0.001; HLA-DRA, Rho=0.592, P<0.001; HLA-DPA1, Rho=0.575, P<0.001), compared to PTGS2 (Rho=0.062, P>0.05), CEACAM8 (Rho=0.016, P>0.05), KIR3DL3 (Rho=0.051, P>0.05), IL13 (Rho=0.008, P>0.05). Moreover, we observed the significant correlations of GLA expression with 47 gene markers among 56 markers investigated in LGG. However, GBM exhibited less GLA/infiltration correlations (32 kinds of related markers). Moreover, we assessed the correlation between GLA level and markers of monocyte, TAM, M1 and M2 macrophages in pan-cancer. Interestingly, among pan-cancer, LGG showed the most significant correlation between GLA with CD86 (P=4.52e-27, **Figure 5A**), CSF1R (P=8.17e-09, **Figure 5B**),CCL2 (P=3.98e-14, **Figure 5C**), CD68 (P=2.37e-34, **Figure 5D**), IL10 (P=3.29e-21, **Figure 5E**), IRF5 (P=8.5e-25, **Figure 6B**), CD163 (P=3.8e-26, **Figure 6D**), VSIG4 (P=9.17e-16, **Figure 6E**) and MS4A4A (P=1.39e-21, **Figure 6F**). However, there were no correlation between GLA expression and NOS2 (P=1.82e-02, **Figure 6A**) or PTGS2 (P=1.57e-01, **Figure 6C**) expression level. Together, this suggested the expression of GLA in LGG may be more predictive of immune infiltration, with potential value for assessment of tumor development.

## Discussion

Immune infiltration is defined as the accumulation of immune cells in blood and tumor tissues and immune effect 20. Glioma metastasis is related to immune system, especially to the function of tumor microenvironment 21. Several studies demonstrated that multiple differentially expressed genes (DEG) in infiltrating immune cells and tumor tissues exerted as the risk factors in prognostic evaluation of glioma patients 22. However, due to the heterogeneity of brain tumors and the blood-brain barrier, the immune microenvironment of LGG remains unclear. It may provide novel insight into dysfunctional immune system by exploring how the tumor microenvironment changes in LGG.

This research presented here provides, as far as we know, the first analysis of GLA level involved in the LGG tumor development using data from TCGA and GEO cohort of patients who have undergone molecular profiling of the malignant tumors. Our assessment of normal brain samples demonstrated significantly lower GLA levels compared to LGG patients. Moreover, it was believable that differences overexpression levels of GLA in LGG tissues contributed to differences in overall survival rate. Interestingly, survival of patients with multiple type of cancers appear to be associated with high GLA expression, indicating that there is a potential interplay between the GLA level and the poor outcome for patients with ACC, LIHC, UVM, STAD and GBM.

Human GLA gene is located at chromosome Xq22 23. GLA mutation results in deletion of GLA protein and the expression of globotriacylsphingolanol (Gb3) and glycoside neurilipids in fibroblasts, where GLA plays a crucial part in affecting heart failure, kidney failure and nervous system disorders 24, 25. In addition, GLA tumor lysate vaccine has been confirmed to induce immune response and inhibit pancreatic cancer development 26. Up to date, several literatures indicated that tumor cells can influence various cells, cytokines and proteases in the microenvironment, especially with infiltrating immune cells 27. The pro-cancer or anti-cancer roles of multiple infiltrated immune cells are comprehensive due to the mutual regulation among various immune cell subtypes 28. In this study, a correlation was found between the expression level of GLA and the level of tissue immune cell infiltration in LGG using the TIMER database. The data indicated that the infiltration of B cells, myeloid dendritic cells, macrophages, monocytes, neutrophils, CD4^+^ T cells and CD8^+^ T cells were significantly related to GLA expression, indicating that GLA was significantly correlated with immunity in LGG tissues.

Moreover, the expression levels of GLA were related to LGG prognosis, instead of GBM prognosis, in multiple immune cells. The association between GLA level and the expression of immune-related markers indicated that GLA regulated immune infiltration in LGG tumor microenvironment. M1 or M2 macrophages are differentiated from tumor-associated macrophages (TAM) via the recruitment and polarization of different cytokines in the microenvironment 29. TAM phenotype in glioma is important for analyzing tumor progression and identifying personalized therapies 30. The results in this study disclosed that there was a weaker association between GLA level and M2 macrophage markers (CD163, VSIG4 and MS4A4A) than M21 macrophage markers, indicating that GLA exerted a crucial role in regulation of TAM polarization. Treg cell is a member of the immunosuppressive CD4^+^ T cell subpopulation. The expression of transcription factor FOXP3 is important for the immune and Treg development in tumors 31. Additionally, Treg cells abundantly secret many kinds of inhibitory cytokines, such as IL-10 and TGF-β 32. Apart from FOXP3, IL-10 and TGF-β expression, Treg cells also express co-inhibitory molecules, which are responsible for Treg cell functional instability, including PD-1, CTLA-4 and VISTA. Among multiple Treg marker molecules, FOXP3 is considered as a molecule regulating essential suppressive mechanism in Treg-like suppressive process 33. In this research, GLA expression was positively associated with levels of Treg-related markers (CCR8 and TGFB1) other than FOXP3.

There are, however, several limitations to our study. First of all, it was necessary to confirm its clinical utility through using more prospective studies and larger LGG cohorts, as we just provided the retrospective analysis based on the data from public databases. Secondly, further *in vitro* and *in vivo* studies are needed to validate GLA's role in LGG tumor immune infiltration. Although the results in this research suggested that GLA played an important role in regulating LGG immune infiltration, the mechanism of underlying regulation requires more experimental studies to validate in future.

Overall, our investigation demonstrated significant overexpression of GLA in LGG malignant tissues. These findings together with the presence of immune cells in LGG tumors displaying high GLA levels may conduce to increased immune infiltration levels help guide immunotherapy in patients with LGG.

## Figures and Tables

**Table 1:** Clinical parameters of the LGG patients

Variable		Number	Percent
Age (year)	<60	15	65.22%
	≥60	8	34.78%
Sex	Male	12	52.17%
	Female	11	47.83%
WHO grade	I	6	26.09%
	II	17	73.91%
	III	0	0.00%
	IV	0	0.00%
Tumor size	< 4 cm	18	78.26%
	≥ 4 cm	5	21.74%
Location	Non-eloquent area	10	43.48%
	Near eloquent area	13	56.52%
Edema	None to minimal	12	52.17%
	Moderate to severe	11	47.83%
Resection degree	Partial to subtotal	10	43.48%
	Gross total	13	56.52%

**Table 2:** Correlation analysis between GLA and immune infiltration markers in LGG and GBM

Description	Gene markers	LGG		GBM	
		Correlation	P	Correlation	P
CD8^+^ T cell	CD8A	0.293	***	0.248	***
	CD8B	0.103	**	0.189	**
T cell	CD3D	0.479	***	0.273	*
	CD2	0.518	***	0.286	***
B cell	CD19	0.294	***	0.003	0.968
	CD79A	0.168	***	0.215	***
Monocyte	CD86	0.45	***	0.376	***
	CSF1R	0.25	***	0.281	***
TAM	CCL2	0.325	***	0.136	0.0943
	CD68	0.502	***	0.39	***
	IL10	0.4	***	0.355	***
M1 macrophage	NOS2	-0.104	**	0.077	0.344
	IRF5	0.431	***	0.226	***
	PTGS2	0.062	0.157	0.084	0.301
M2 macrophage	CD163	0.442	***	0.29	***
	VSIG4	0.344	***	0.358	***
	MS4A4A	0.403	***	0.411	***
Neutrophils	CEACAM8	0.016	0.723	-0.138	0.008
	ITGAM	0.361	***	0.211	**
	CCR7	0.391	***	0.312	***
Natural killer cell	KIR2DL1	0.138	***	0.212	***
	KIR2DL3	0.182	***	-0.014	0.866
	KIR2DL4	0.366	***	0.142	0.081
	KIR3DL1	0.139	***	0.101	0.214
	KIR3DL2	0.139	***	0.031	0.699
	KIR3DL3	0.051	0.25	-0.042	0.61
	KIR2DS4	0.18	***	0.063	0.438
Dendritic cell	HLA-DPB1	0.572	***	0.362	***
	HLA-DQB1	0.468	***	0.181	**
	HLA-DRA	0.592	***	0.338	***
	HLA-DPA1	0.575	***	0.331	***
	CD1C	0.294	***	0.286	***
	NRP1	0.426	***	0.33	***
	ITGAX	0.377	***	0.157	0.0519
Th1	TBX21	0.434	***	0.188	**
	STAT4	-0.005	0.908	0.183	**
	STAT1	0.557	***	-0.013	0.876
	IFNG	0.265	***	0.094	0.247
	TNF	-0.015	0.734	0.155	0.565
Th2	GATA3	0.452	***	0.143	0.0787
	STAT6	0.262	***	0.086	**
	STAT5A	0.422	***	0.245	0.289
	IL13	0.008	0.858	-0.094	0.246
Tfh	BCL6	0.058	0.185	-0.1	0.221
	IL21	0.114	***	0.116	0.153
Th17	STAT3	0.525	***	-0.046	0.57
	IL17A	0.089	*	-0.007	0.935
Treg	FOXP3	0.053	0.233	0.156	0.0546
	CCR8	0.25	***	0.228	**
	STAT5B	0.044	0.319	-0.127	0.116
	TGFB1	0.379	***	0.164	*
T cell exhaustion	PDCD1	0.468	***	0.348	***
	CTLA4	0.351	***	0.28	***
	LAG3	0.265	***	0.209	***
	HAVCR2	0.479	***	0.368	***
	GZMB	0.362	***	0.201	*

*P < 0.05, **P < 0.01, ***P < 0.001.

## Abbreviations

GLA: Galactosidase alpha
LGG: low-grade glioma
GEO: gene expression omnibus
TCGA: the Cancer Genome Atlas
CNS: central nervous system
PFS: progression-free survival
NK: natural killer
DC: dendritic cells
GBM: glioblastoma
OS: overall survival
NCBI: the National Center of Biotechnology information
GEPIA2: Gene expression Profiling Interactive Analysis 2
TIMER: the Tumor Immune Estimation Resource
cor: correlation
THCA: thyroid carcinoma
LUAD: lung adenocarcinoma
ACC: adrenocortical carcinoma
LIHC: liver hepatocellular carcinoma
UVM: uveal melanoma
STAD: stomach adenocarcinoma
Gb3: globotriacylsphingolanol
TAM: tumor-associated macrophages
DEG: differentially expressed genes
